# In a rat model of bypass DuraGraft ameliorates endothelial dysfunction of arterial grafts

**DOI:** 10.1038/s41598-024-66056-3

**Published:** 2024-07-02

**Authors:** Shuo Lian, Sivakkanan Loganathan, Tobias Mayer, Patricia Kraft, Alex Ali Sayour, Adrian-Iustin Georgevici, Gábor Veres, Matthias Karck, Gábor Szabó, Sevil Korkmaz-Icöz

**Affiliations:** 1 Laboratory of Cardiac Surgery, Department of Cardiac Surgery, University Hospital Heidelberg, INF 326, 69120 Heidelberg, Germany; 2 Department of Cardiac Surgery, University Hospital Halle (Saale), 06120 Halle, Germany; 3 Heart and Vascular Center, Semmelweis University, Budapest, 1122 Hungary; 4 Department of Anesthesiology, St. Josef Hospital, Ruhr-University Bochum, 44791 Bochum, Germany

**Keywords:** DuraGraft®, Graft, Ischemia/reperfusion injury, Endothelial dysfunction, Preservation solution, Diseases, Medical research

## Abstract

Coronary artery bypass surgery can result in endothelial dysfunction due to ischemia/reperfusion (IR) injury. Previous studies have demonstrated that DuraGraft helps maintain endothelial integrity of saphenous vein grafts during ischemic conditions. In this study, we investigated the potential of DuraGraft to mitigate endothelial dysfunction in arterial grafts after IR injury using an aortic transplantation model. Lewis rats (n = 7–9/group) were divided in three groups. Aortic arches from the control group were prepared and rings were immediately placed in organ baths, while the aortic arches of IR and IR + DuraGraft rats were preserved in saline or DuraGraft, respectively, for 1 h before being transplanted heterotopically. After 1 h after reperfusion, the grafts were explanted, rings were prepared, and mounted in organ baths. Our results demonstrated that the maximum endothelium-dependent vasorelaxation to acetylcholine was significantly impaired in the IR group compared to the control group, but DuraGraft improved it (control: 89 ± 2%; IR: 24 ± 1%; IR + DuraGraft: 48 ± 1%, *p* < 0.05). Immunohistochemical analysis revealed decreased intercellular adhesion molecule-1, 4-hydroxy-2-nonenal, caspase-3 and caspase-8 expression, while endothelial cell adhesion molecule-1 immunoreactivity was increased in the IR + DuraGraft grafts compared to the IR-group. DuraGraft mitigates endothelial dysfunction following IR injury in a rat bypass model. Its protective effect may be attributed, at least in part, to its ability to reduce the inflammatory response, oxidative stress, and apoptosis.

## Introduction

Coronary artery bypass grafting (CABG) is a major surgical procedure that involves the use of autologous grafts, such as arteries or veins, to bypass partially or completely obstructed coronary arteries^1^. Graft failure often occurs due to vascular ischemia/reperfusion (IR) injury, which can happen during the harvesting process. However, the preservation solution in which the graft is temporarily immersed also plays a crucial role in endothelial dysfunction^2,3^. Endothelial dysfunction refers to an imbalance in the production of vasodilators and vasoconstrictors, an excess of reactive oxygen species (ROS), diminished production/availability of nitric oxide (NO), as well as upregulation of pro-inflammatory cell adhesion molecules^4^. Physiological saline or autologous whole blood are the most common solutions used to preserve the endothelial integrity of saphenous veins (SVGs) and free arterial grafts during CABG. However, graft failure remains a common complication of CABG^5^. Therefore, novel strategies are urgently needed to decrease preservation injury and improve graft function^6^.

DuraGraft is the only clinically approved storage solution that can protect SVGs. The solution’s composition includes physiological salts, antioxidants, L-glutathione, L-ascorbic acid, and L-arginine, which serves a substrate for NO synthase found in endothelial cells^7,8^. In a retrospective study, it was found that the use of DuraGraft during surgery significantly reduced clinical complications following CABG procedures. These included a 45% decrease in non-fatal myocardial infarction (*p* < 0.0001), a 35% decrease in repeat revascularization (*p* = 0.037), and a 19% decrease in major adverse cardiovascular events (*p* = 0.005)^9^. Additionally, studies have demonstrated that DuraGraft is effective in maintaining the structural integrity and viability of isolated pig mammary veins and human saphenous vein segments under ischemic conditions, compared to saline, blood, and buffered solutions^5^. Arterial grafts are considered better conduits than SVGs in CABG^10^. Recently, Aschacher et al. demonstrated that DuraGraft reduces oxidative stress and improves cellular integrity in radial artery grafts used for CABG^11^. These studies have nevertheless examined the effect of DuraGraft during the ischemic preservation phase. However, few studies have investigated the effects of DuraGraft on arterial grafts submitted to cold ischemia followed by warm reperfusion in an in vivo model.

This study analyzes the effect of DuraGraft on endothelial dysfunction induced by cold ischemia followed by blood reperfusion in a rat bypass model.

## Results

### Effect of intraoperative preservation with DuraGraft on aortic contractile responses

**Figure 1:**
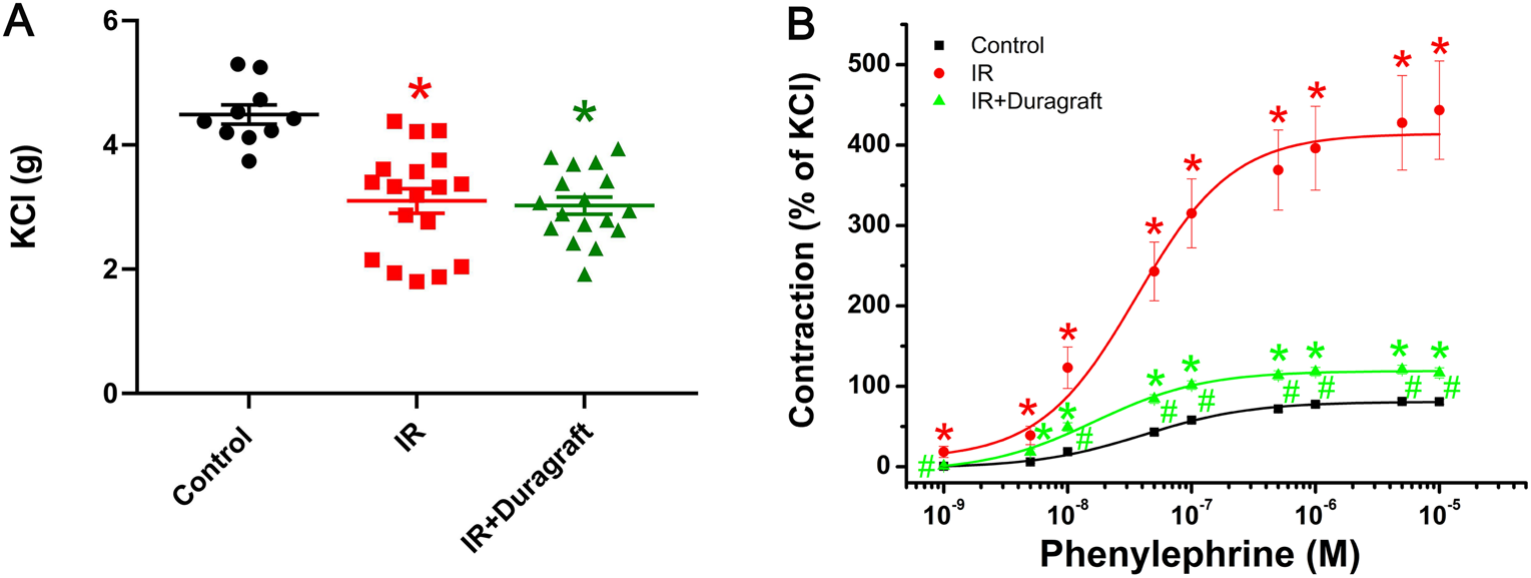
Effect of DuraGraft on contractile responses after cold ischemia followed by blood reperfusion. Contractile responses to (**A**) high potassium-induced depolarization (control: *n* = 14 rings from 7 rats; IR: *n* = 18 rings from 9 rats; IR + Duragraft: *n* = 17 rings from 9 rats) and (**B**) phenylephrine (expressed as a percentage of the maximum contraction induced by 80 mM potassium chloride (KCl)) (control: *n* = 14 rings from 7 rats; IR: *n* = 18 rings from 9 rats; IR + Duragraft: *n* = 17 rings from 9 rats). The results are presented as mean ± SEM. *p < 0.05 versus control and ^#^*p* < 0.05 versus IR.

In response to high potassium (K^+^)-induced depolarization, contraction was significantly reduced in the IR group compared to controls. However, the use of DuraGraft did not show any significant effect on the contractile responses (Fig. 1A). Conversely, the increased contractility to phenylephrine observed in the IR group compared to controls was significantly decreased after preservation of aortic rings with DuraGraft (Fig. 1B). The pD_2_ values, which indicate the sensitivity of the aorta to phenylephrine, were significantly lower in the IR group compared to controls but similar between the IR + DuraGraft and the control groups (Table 1).

**Table 1:** Quantitative analysis of vascular function: effect of DuraGraft after cold ischemia followed by blood reperfusion in the aorta. Contractile responses to phenylephrine (control, n = 14 rings from 7 rats; IR, n = 18 rings from 9 rats; IR + Duragraft, n = 17 rings from 9 rats) were expressed as gram and as a percentage of the maximum contraction induced by 80 mM potassium chloride (KCl) (control, n = 14 rings from 7 rats; IR, n = 18 rings from 9 rats; IR + Duragraft, n = 17 rings from 9 rats). Maximum relaxation to acetylcholine (control, n = 14 rings from 7 rats; IR, n = 18 rings from 9 rats; IR + Duragraft, n = 17 rings from 9 rats) and sodium nitroprusside (SNP) (control, n = 14 rings from 7 rats; IR, n = 18 rings from 9 rats; IR + Duragraft®, n = 17 rings from 9 rats) were expressed as a percentage of the contraction induced by phenylephrine.

	Control	IR	IR + Duragraft
KCl (g)	4.49 ± 0.13	3.10 ± 0.20*	3.03 ± 0.14*
Phenylephrine (%)	77.0 ± 4.0	98.2 ± 4.7*	108.2 ± 3.2*^#^
pD_2_ to phenylephrine	7.15 ± 0.09	6.76 ± 0.09*	6.94 ± 0.09
R_max_ to acetylcholine (%)	88.7 ± 2.2	23.7 ± 0.8*	48.3 ± 0.8*^#^
pD_2_ to acetylcholine	6.20 ± 0.19	6.64 ± 0.28	5.52 ± 0.19^#^
R_max_ to SNP (%)	100.0 ± 0.0	100.0 ± 0.0	100.0 ± 0.0
pD_2_ to SNP	8.50 ± 0.30	8.23 ± 0.24	8.87 ± 0.29

R_max_ indicates maximum relaxation and pD_2_, negative logarithm of the corresponding half-maximal response (EC_50_). Results are presented as mean ± standard error of the mean (SEM). *p < 0.05 versus control and ^#^*p* < 0.05 versus IR.

### Effect of intraoperative preservation with DuraGraft on aortic endothelial function

**Figure 2:**
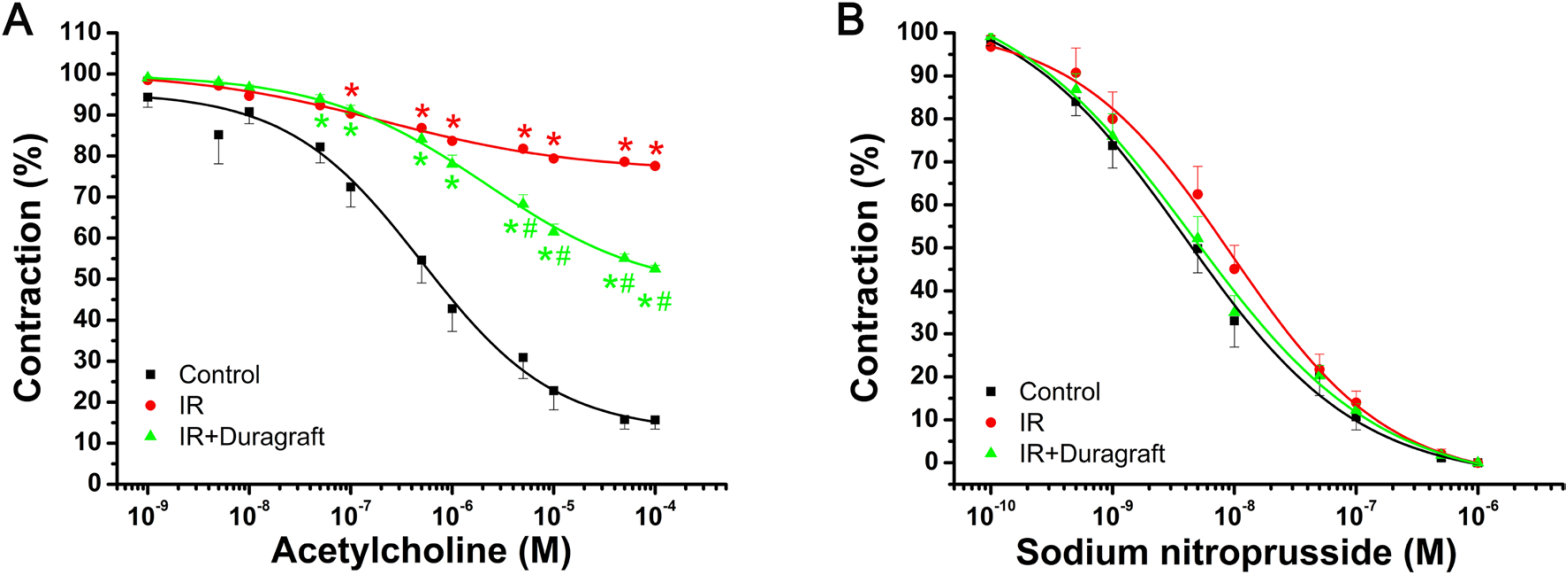
Effect of DuraGraft on relaxation responses after cold ischemia followed by blood reperfusion. (**A**) Acetylcholine-induced endothelium-dependent vasorelaxation (control: *n* = 14 rings from 7 rats; IR: *n* = 18 rings from 9 rats; IR + Duragraft: *n* = 17 rings from 9 rats) and (**B**) sodium nitroprusside-induced endothelium-independent vasorelaxation (control: *n* = 14 rings from 7 rats; IR: *n* = 18 rings from 9 rats; IR + Duragraft: *n* = 17 rings from 9 rats) in aortic rings. The results are presented as mean ± SEM. *p < 0.05 versus control and ^#^*p* < 0.05 versus IR.

All aortic rings that were pre-contracted with phenylephrine showed concentration-dependent relaxation to acetylcholine at concentrations of 10^–9^ to 10^–4^ M (Fig. 2A). Vasorelaxation induced by acetylcholine was significantly lower in the IR group compared to controls, but it was improved in aortic rings preserved with DuraGraft (Fig. 2A). The sensitivity of the aorta to acetylcholine, indicated by pD_2_ values, was significantly decreased in the IR + DuraGraft group compared to the IR aortas (Table 1).

### Effect of intraoperative preservation with DuraGraft on aortic smooth muscle relaxation

In all phenylephrine pre-contracted aortic rings, concentration-dependent relaxation was elicited by sodium nitroprusside (10^–10^–10^–5^ M) (Fig. 2B). There were no significant changes observed among the experimental groups in terms of maximum relaxation responses or pD_2_ values to sodium nitroprusside (Table 1**; **Fig. 2B).

### Effect of intraoperative preservation DuraGraft on aortic caspase-3, caspase-8, intercellular adhesion molecule (ICAM)-1, 4-hydroxy-2-nonenal, and platelet and endothelial cell adhesion molecule (PECAM)-1 immunoreactivity

**Figure 3:**
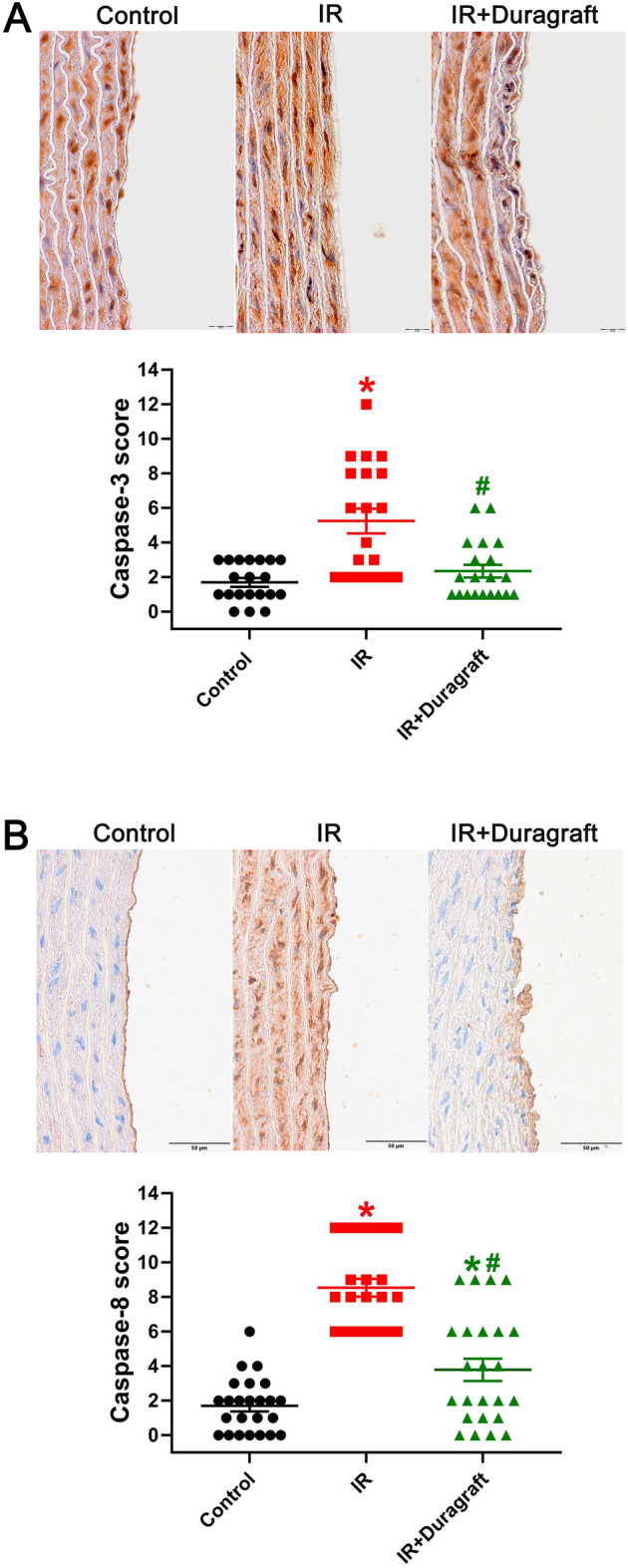
Effect of DuraGraft on caspase-3 and caspase-8 immunoreactivity after cold ischemia followed by blood reperfusion. Representative micrographs of (**A**) caspase-3 (× 400, scale: 10 μm) (*n* = 20 pictures from 6 rats) and (**B**) caspase-8 (× 400, scale: 50 μm) (*n* = 24 pictures from 6 rats) followed by semi-quantitative evaluation of aortic rings. A blinded examination of two to four randomized non-overlapping fields per aorta per rat was conducted by two examiners. The results are represented as mean ± SEM. *p < 0.05 versus control and ^#^*p* < 0.05 versus IR.

**Figure 4:**
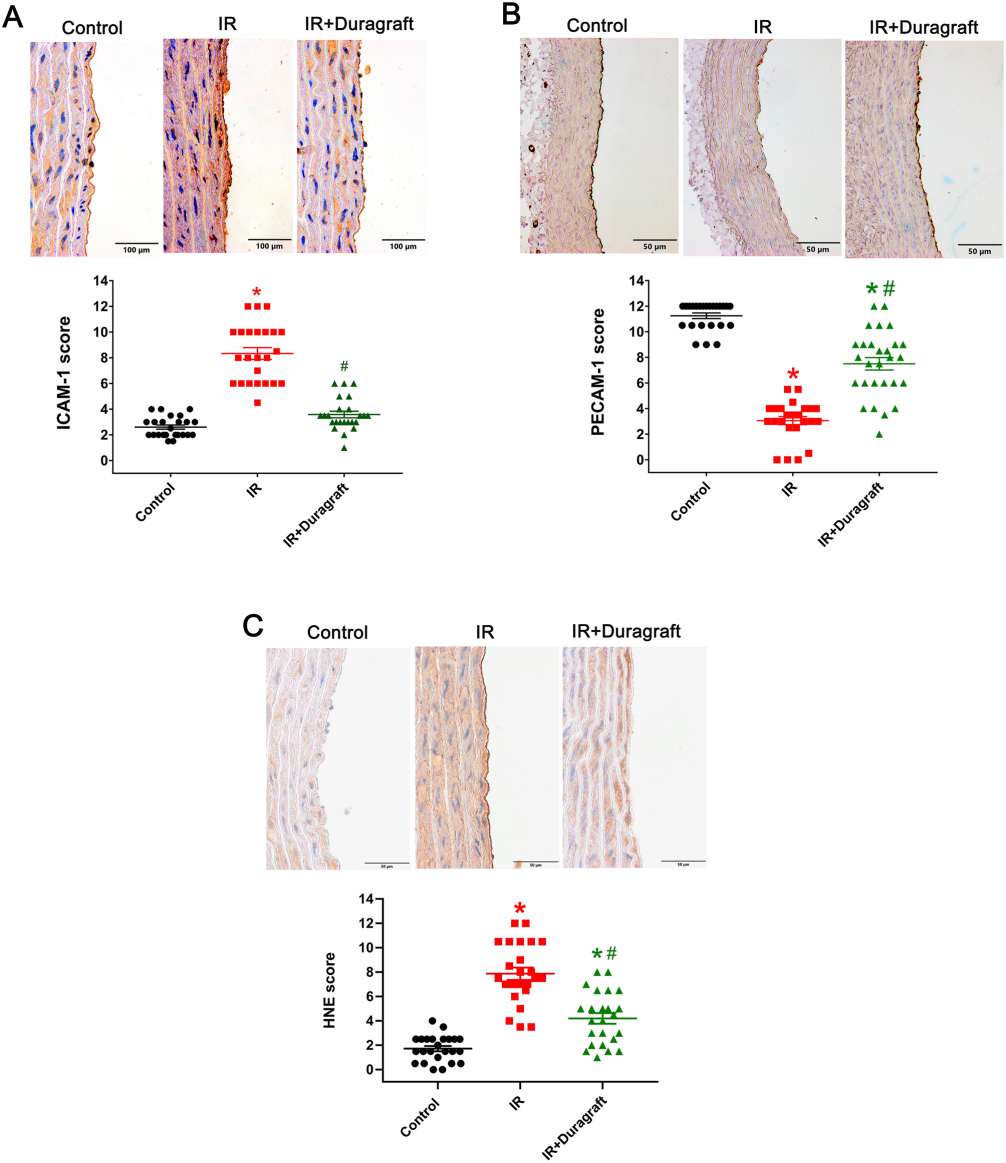
Effect of DuraGraft on intercellular adhesion molecule (ICAM)-1, platelet and endothelial cell adhesion molecule (PECAM)-1, and 4-hydroxy-2-nonenal (HNE) immunoreactivity after cold ischemia followed by blood reperfusion. Representative micrographsof (**A**) ICAM-1 (× 400, scale: 100 μm), (**B**) PECAM-1 (× 400, scale 50 µm) and (C) HNE (× 400, scale: 50 μm) followed by semi-quantitative evaluation of aortic rings. A blinded examination of two to four randomized non-overlapping fields per aorta per rat was conducted by two examiners. The results are represented as mean ± SEM. *p < 0.05 versus control and ^#^*p* < 0.05 versus IR. *n* = 24 pictures from 6 rats/group.

Our findings showed a significant increase in the immunoreactivity of caspase-3 and caspase-8, essential regulators of the apoptotic response, in the IR group compared to the control group (Fig. 3). However, preservation of aortas with DuraGraft solution led to a decrease in caspase3 and caspase-8 immunoreactivity (Fig. 3). Furthermore, the increased expression of ICAM-1, which is responsible for regulating leukocytes recruitment from circulation to sites of inflammation, and 4-hydroxy-2-nonenal, a marker of oxidative stress, observed in the IR aortas compared to controls, was significantly reduced after preservation with DuraGraft (Fig. 4A and 4C). Additionally, a significant decrease in endothelial PECAM-1 immunoreactivity was observed in the IR aortas compared to controls, which was increased following preservation with DuraGraft (Fig. 4B).

## Discussion

Our findings suggest that the preservation of arterial grafts using DuraGraft, a novel inhibitor of endothelial damage, mitigates endothelial dysfunction and reduces enhanced smooth muscle contraction induced by agonists after cold ischemia and warm reperfusion injury. The mechanism underlying the positive effect of DuraGraft preservation on endothelial function and smooth muscle contraction involves a decrease in ICAM-1 expression, an increase in PECAM-1 expression, a reduction in oxidative stress, and a decrease in caspase-mediated apoptosis.

CABG is a surgical technique that involves using autologous arteries or SVGs to bypass occlusion and restore blood supply to the heart muscle^12,13^. Unfortunately, conventional storage solutions used during CABG surgery do not adequately protect the endothelium. Consequently, post-procedural lumen loss can occur following CABG due to functional and structural changes in endothelial cells, among other factors^12,14^. In our study, we confirmed that cold ischemia followed by reperfusion with blood leads to endothelial dysfunction and impairs contractile responses in arterial grafts. These impairments are attributed, in part, to inflammation, oxidative stress, and cell apoptosis, which are crucial factors in IR-induced vascular damage^12,14,15^. Moreover, our findings revealed significant changes in the levels of intercellular adhesion molecules, especially ICAM-1 and PECAM-1, known to contribute to the inflammatory mechanisms in blood vessels. Additionally, we observed modifications in the levels of 4-hydroxy-2-nonenal, a protein biomarker of oxidative stress, as well as caspase-3 and caspase-8, important regulators of apoptosis. Therefore, novel treatments are required to protect vascular grafts against IR injury, potentially improving long-term outcomes following CABG^6^.

In this context, DuraGraft, an endothelial damage inhibitor, has shown promising results in preclinical and clinical investigations in protecting the integrity and function of SVG’s endothelium during ischemic storage^16^. A retrospective study found that treatment of SVGs with DuraGraft during the CABG procedure was linked to lower rates of major adverse cardiac events and repeat revascularization compared to the use of heparinized saline in a cohort of 2436 patients^9^. In a recent study, we demonstrated that DuraGraft improves both endothelial and contractile dysfunction resulting from in vitro vascular injury caused by IR, without the involvement of leukocytes^17^. Building upon the promising findings from our in vitro investigation^17^, we conducted an experimental study using a clinically relevant in vivo rat model to compare the impact of DuraGraft and saline on arterial vascular damage. Acetylcholine binds to muscarinic receptors on endothelial cells to induce NO release, which exhibits a crucial role in vascular tone, local blood flow, leukocyte-endothelial cell interactions, and platelet aggregation^18^. Our study showed that DuraGraft protects against endothelial dysfunction. This is demonstrated by the enhancement of endothelium-dependent vasorelaxation to acetylcholine and attenuation of exaggerated agonist-induced smooth muscle contraction following IR injury. To evaluate the loss of endothelial cells within the grafts, we performed PECAM-1 immunoreactivity. Our results demonstrated that the decreased immunoreactivity of PECAM-1, a marker indicating the presence of endothelial cells, caused by IR injury, was increased by DuraGraft treatment. These observations are in line with previous studies that have shown DuraGraft to preserve the functionality and integrity of endothelial and intimal cells, prevent DNA damage, and reduce cell death in radial artery grafts used for CABG^11^. The authors demonstrated in their study that DuraGraft decreases ROS production and inhibits progressive neo-intimal formation by downregulating transforming growth factor (TGF)-β-induced vascular endothelial growth factor (VEGF) cellular over-proliferation^11^. Additionally, they showed a direct positive effect on protective markers, such as heme oxygenase-1 and AKT/endothelial NO synthase pathway^11^. Ischemia followed by reperfusion is known to elicit a widespread inflammatory response, which is typically marked by the upregulation of adhesion molecules. The expression of ICAM-1 can be induced by various cytokines (such as interleukin-1β, tumor necrosis factor-α, and interferon (IFN)-γ)^19^, as well as by cytokine-independent stimuli such as free radicals and hypoxia, including reactive and nitrogen species^20^. Our study demonstrated that DuraGraft resulted in a significant decrease in ICAM-1 immunoreactivity in aortic rings subjected to cold ischemia followed by warm reperfusion injury. Furthermore, we performed immunohistochemistry for HNE, an indicator of oxidative stress, based on the role of oxidative stress during IR injury. Our results showed that DuraGraft lowered the increased 4-hydroxy-2-nonenal expression in aortic rings. Additionally, DuraGraft reduced high caspase-3 and caspase-8 immunoreactivity in the arterial grafts, which are key regulators of the apoptotic response, after IR injury.

In a previous study, we have also shown that the storage of grafts with saline or heparinized blood solution (maximum relaxation to acetylcholine: 34 ± 6% or 29 ± 4%) is unable to protect the endothelium against cold ischemia and warm reperfusion injury in rats^21^. In another study, our findings support that the N-acetyl-histidine containing iron chelators–enriched and amino-acid fortified TiProtec solution appears to be superior to saline and custodiol (maximum relaxation to acetylcholine: 46 ± 7% vs. 26 ± 5% and 24 ± 5%) as a preservation solution for arterial storage in a rat model of bypass^22^. Consistent with the results of the present study, the maximum relaxation to acetylcholine in the saline group was 24 ± 1%, whereas with DuraGraft it was 48 ± 1%, indicating similar protective effect of TiProtect and DuraGraft on arterial grafts in a rodent model of revascularization. Additionally, the reduced immunoreactivity of PECAM-1 (CD31), a marker indicating the presence of endothelial cells, resulting from ischemia/reperfusion injury, was ameliorated by TriProtect^22^, as observed in the present study.

The present study has some limitations. First, it should be noted that the vessel wall structure of the aortic tissue differs from that of human internal mammary or radial artery grafts. Therefore, caution should be exercised when interpreting our results in the context of those grafts. Second, although vascular leakage is an important feature in several diseases, including IR injury, endothelial permeability was not measured in this study.

In summary, our study presents novel experimental evidence demonstrating that preservation of arterial grafts with DuraGraft alleviates endothelial dysfunction and attenuates agonist-induced increased smooth muscle contraction following cold ischemia and blood reperfusion in rats. Our findings suggest that the protective effect of DuraGraft can be attributed, at least in part, to its ability to reduce oxidative stress and apoptosis, decrease ICAM-1 expression, and increase PECAM-1 expression. However, additional studies are necessary to fully elucidate the mechanisms underlying the protective effects of DuraGraft.

## Methods

### Animals and humane care

Isogenic male Lewis rats weighing between 250 and 300 g were obtained from Janvier Labs, Saint Berthevin, France, and housed in the Division of Laboratory Animal Resources at Heidelberg University, Germany. Before experiments, the rats were acclimatized for at least 7 days and given standard rodent fodder and water ad libitum. The animal procedures in this study followed the "Principles of Laboratory Animal Care" published by the National Society for Medical Research and the "Guide for the Care and Use of Laboratory Animals" developed by the Institute of Laboratory Animal Resources and published by the National Institutes of Health in 2011 (8th edition). Ethical approval for this study was obtained from the Ethical Committee of the Regional Council of Karlsruhe, Germany (G280/20). The animal experiments were conducted in accordance with the ARRIVE (Animals in Research: Reporting in Vivo Experiments) guidelines.

### Experimental group

**Figure 5:**
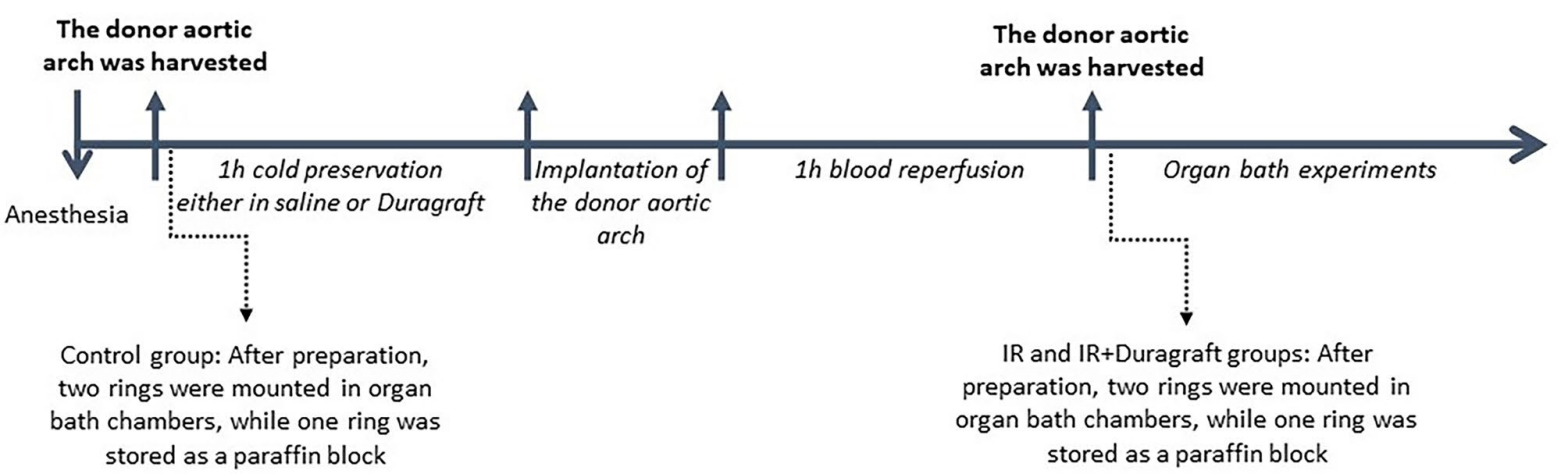
Sequential outline of the experimental procedure. After anesthesia, the donor aortic arch was harvested for all experimental groups. In the control group, after preparation, two rings were mounted in organ bath chambers, while one ring was stored as a paraffin block. For the IR and IR + Duragraft groups, the aortic arch was preserved for 1 h at 4 °C either in saline or Duragraft, respectively. Then, the donor aortic arch was implanted into the recipient. After 1 h of blood reperfusion, the donor aortic arch was harvested, and two prepared rings were mounted in organ bath chambers, while one ring was stored as a paraffin block. The rings were excluded from the organ bath experiments analysis if there was a technical problem, and they were considered damaged.

The animals were randomly assigned to three groups: control (n = 7 rats), IR (n = 9 rats), and IR + DuraGraft (n = 9 rats). In the control group, the aortic arches were explanted, and the aortic rings were prepared and immediately placed in organ baths. In the IR- and IR + DuraGraft groups, the donor aortic arches were explanted, stored in cold physiological saline or DuraGraft solution (Somahlution, Jupiter, FL, United States), respectively, for 1 h before heterotopic aorta transplantation. Following 1 h of reperfusion, the aortic arches were removed and placed in chambers of organ bath for ex vivo measurement of vascular function in the grafts. The experimental protocol is shown in Fig. 5**.**

### Heterotopic aorta transplantation

As we previously described^23^, anesthesia was initially induced by placing the donor male Lewis rats in a chamber filled with 3% isoflurane gas and was maintained by inhaling 1.75–2.5% in oxygen through a connected tube. Heparin (400 IU/kg, Ratiopharm GmbH, Ulm, Germany) was administered through the inferior vena cava, and the brachiocephalic, left common carotid artery, and left subclavian artery were ligated. While the aortic arches obtained from the control group were explanted, the aortic rigs were prepared, cut into rings, and immediately placed in organ bath chambers, those from the IR and IR + DuraGraft groups were preserved in saline or DuraGraft, respectively, for a duration of 1 h. The recipient rats were anesthetized and then heparinized following the same procedure as describe before. Two ends to side anastomoses were used to transplant the donor aortic arch into the recipient aorta.

### Ex vivo organ bath experiments

As we previously described^17,24^, the aortic arch was explanted for the control group, while for the IR and IR + DuraGraft groups, the implanted graft was harvested after 1 h of in vivo blood reperfusion. The harvested graft was then placed in Krebs–Henseleit solution (KHL) containing the following components: 118 mM NaCl, 4.7 mM KCl, 1.2 mM KH_2_PO_4_, 1.2 mM MgSO_4_, 1.77 mM CaCl_2_, 25 mM NaHCO_3_ and 11.4 mM glucose). Subsequently, the aortic arch was carefully dissected and cut into three rings, each measuring 3–4 mm in width. Two rings were then mounted on the hooks of isolated organ baths containing 30 ml KHL at 37 °C while the remaining ring was used for immunohistochemical staining. The baths were continuously supplied with a gas mixture of 95% O_2_ and 5% CO_2_. To maintain a passive tension of 2 g, the rings were stretched and allowed to equilibrate for a duration of 1 h, with KHL being replaced every 30 min to prevent the presence of interfering metabolites. Throughout this period, adjustments were periodically made to the tension, maintaining it at 2 g. Following equilibration, to verify tissue viability, 80 mM potassium chloride (KCl) was used. Then, the rings were pre-contracted using an α-adrenergic receptor agonist, phenylephrine (10^–9^–10^–5^ M). Subsequently, relaxation responses were assessed by adding increasing concentrations of acetylcholine (10^–9^–10^-4^ M) and sodium nitroprusside (10^–10^–10^–5^ M). The relaxation responses were expressed as a percentage of the contraction induced by phenylephrine. The half-maximal response values (EC_50_) were obtained from sigmoidal equation fitting of individual concentration–response curves to phenylephrine, acetylcholine, or sodium nitroprusside, and the vascular sensitivity was expressed as pD_2_ (− logEC_50_).

### Immunohistochemistry for caspase-3, caspase-8, ICAM-1, PECAM-1, and 4-hydroxy-2-nonenal

As we previously reported^17,24^, immunohistochemical analysis was performed on aortic segments that were fixed in a 4% buffered paraformaldehyde solution and embedded in paraffin. Thin sections of 4 µm thickness were cut from the tissue blocks and subjected to various treatments to unmask the antigenic epitopes. Primary antibodies against ICAM-1 (1:50, ab171123, host species: mouse, monoclonal, Thermo Sientific, UK), PECAM-1 (1:5000, ab182981, host species: rabbit, monoclonal, Abcam, Berlin, Germany), HNE (1:1000, ab46545, host species: rabbit, polyclonal, Abcam, Berlin, Germany), caspase-3 (1:1000, #9662, host species: rabbit, polyclonal, Cell Signaling Technology, Massachusetts, USA), and caspase-8 (1:5000, NB100-56,116, host species: rabbit, polyclonal, Bio-Techne, LTD, UK) were applied to the sections and incubated overnight at 4 °C. The samples were then treated with a biotinylated secondary antibody and visualized using the ABC reagent and 3,3' diaminobenzidine (DAB substrate). After cleaning, the sections were mounted and counterstained with hematoxylin. Semi-quantitative immunohistochemistry analysis was conducted using a typical light microscope and CellSens software (Olympus Soft Imaging Solutions GmbH, Münster, Germany). The analysis was based on the staining distribution patterns, which were observed along the endothelium for ICAM-1 and PECAM-1 or within the smooth muscle tissue for 4-hydroxy-2-nonenal. Additionally, the intensity of the staining, ranging from 0 to 12, was evaluated. The staining distribution patterns and intensity of staining were assessed in a blinded manner by examining two to four randomized non-overlapping fields per aorta per rat.

### Statistical data analysis

The data was presented as mean ± standard error of the mean (SEM). Statistical analyses were performed using GraphPad Prism 8.0 software (GraphPad Software, Inc., CA, USA). The normality of data distribution for contractility to KCl, pD_2_ values, and histological data was evaluated using the Shapiro–Wilk normality test. For normally distributed data, multiple comparisons were conducted using one-way ANOVA followed by Tukey’s post-hoc test. In cases where the data did not follow a normal distribution, the nonparametric Kruskal–Wallis test followed by Dunn’s post-hoc test was employed. In the analysis of cumulative concentration–response curves to phenylephrine, acetylcholine, and sodium nitroprusside, a two-factor mixed ANOVA was used for multiple comparisons, followed by Tukey’s post-hoc test. Statistical significance was defined as *p* < 0.05.

## Data Availability

The datasets generated ad/or analysed during the current study are available from the corresponding author, upon reasonable request.
